# Atmospheric- and Low-Level Methane Abatement *via* an Earth-Abundant Catalyst

**DOI:** 10.1021/acsenvironau.1c00034

**Published:** 2021-12-29

**Authors:** Rebecca J. Brenneis, Eric P. Johnson, Wenbo Shi, Desiree L. Plata

**Affiliations:** † Ralph M. Parsons Laboratory, School of Engineering, Massachusetts Institute of Technology, 15 Vassar Street, Cambridge, Massachusetts 02139-4307, United States; ‡ School of Engineering and Applied Sciences, Yale University, 17 Hillhouse Avenue, New Haven, Connecticut 06520, United States

**Keywords:** zeolite, methane, ion exchange, low-temperature processing, direct air capture, greenhouse gas conversion

## Abstract

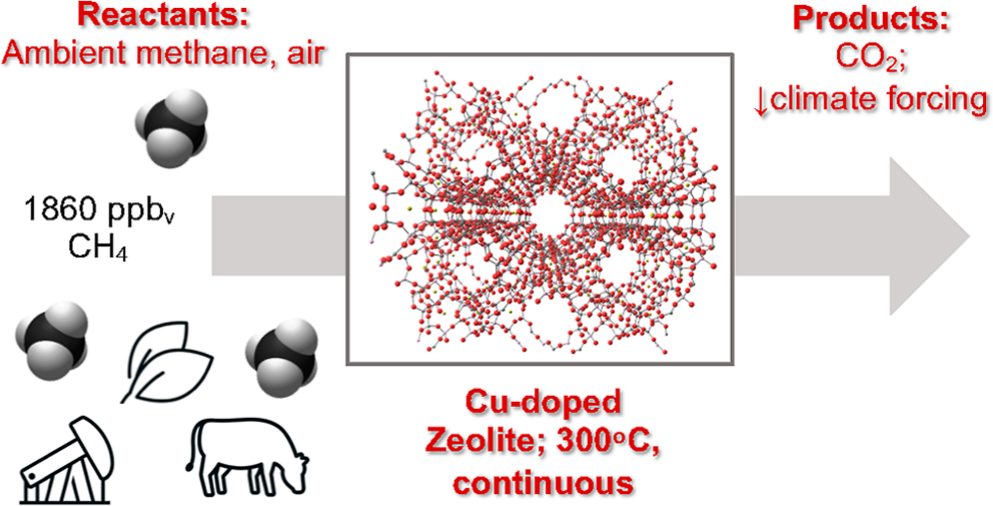

Climate action scenarios that limit changes in global temperature to less than 1.5 °C require methane controls, yet there are no abatement technologies effective for the treatment of low-level methane. Here, we describe the use of a biomimetic copper zeolite capable of converting atmospheric- and low-level methane at relatively low temperatures (*e.g.*, 200–300 °C) in simulated air. Depending on the duty cycle, 40%, over 60%, or complete conversion could be achieved (*via* a two-step process at 450 °C activation and 200 °C reaction or a short and long activation under isothermal 310 °C conditions, respectively). Improved performance at longer activation was attributed to active site evolution, as determined by X-ray diffraction. The conversion rate increased over a range of methane concentrations (0.00019–2%), indicating the potential to abate methane from any sub-flammable stream. Finally, the uncompromised catalyst turnover for 300 h in simulated air illustrates the promise of using low-cost, earth-abundant materials to mitigate methane and slow the pace of climate change.

## Introduction

The Intergovernmental Panel on Climate Change and the United Nations have recently published calls to action to reduce atmospheric methane emissions in order to slow the rate of global temperature change as quickly as possible.^1,2^ The reason methane is uniquely well suited to bring urgently needed reductions in climate warming rates is because methane has a pronounced radiative forcing effect in the near term: instantaneously, methane is 120 times as powerful a warmer as CO_2_ on a per-mass basis and exhibits a 28–34-fold greater global warming potential even after 100 years.^3,4^ By 2030, methane will do as much damage as CO_2_ in spite of methane’s much lower atmospheric abundance (1.85 ppmv CH_4_*vs* 417 ppmv CO_2_).^3,5,6^ Considering these temporally dynamic effects, reducing atmospheric methane levels presents a unique opportunity for a rapid and large impact on climate. For example, restoring atmospheric concentrations to pre-industrial levels (ca. 0.76 ppmv) would reduce radiative forcing by as much as 16% within only 10–20 years.^7,8^ However, methane in the atmosphere is steadily increasing, and the time frame for meeting warming reduction goals is narrowing.^5,7^

Historically, the approach to reducing methane emissions at concentrated sources (greater than 4%) has been to flare the stream to CO_2_. Emergent efforts to reduce primary emissions include finding and stopping leaks from fossil energy systems,^9^ leveraging landfill and other anaerobic gas for energy,^10^ or reducing enteric fermentation in animal husbandry practices.^11,12^ However, the dominant sources of methane to the atmosphere are both distributed and diffuse (*e.g.*, 465–607 Tg yr^–1^; 81–83% globally).^5^ As these sources are spatially dispersed, temporally transient, and often below flammability limits, engineering solutions have been uneconomical or intractable. Strategies to reduce atmospheric methane should be able to act on atmospheric levels anywhere or be co-located with enrichments in the presence of oxygen at or near ambient conditions.

Biological systems have evolved to achieve a similar chemistry: to react low-level methane at relatively low temperatures in sub-oxic environments. In particular, methanotrophs utilize methane monooxygenase (MMO) enzymes to metabolize available methane to methanol.^13,14^ Existing in multiple forms but with a highly conserved active site, MMOs feature high-spin reactive oxygen capable of activating C–H bonds and a redox-controlled “breathable” pore structure that directs reagents to prevent errant diffusion that could lead to “overoxidation” to CO_2_.^13−15^ Over the last decade, attempts to replicate this chemistry *via* transition metals and zeolites have been promising.^15−24^ The zeolite pore structures enhance catalytic reactions by placing metal oxides into unfavorably high energetic configurations that geometrically constrain gaseous reactants into contact through nanoscale channels. Most efforts have focused on methane-to-methanol conversion for fuel and chemical production under industrial conditions,^25,26^ but these efforts have been stymied by a lack of selectivity, overoxidation, and poor economics of methanol separation and use. Further, in an attempt to reduce overoxidation to CO_2_, researchers have employed extreme oxygen levels (*e.g.*, 100%) in a two-step process of catalyst oxidation followed by reaction with high levels of methane, which is irrelevant to deployment in the environment and also not representative of the evolutionary optimum of MMOs (*i.e.*, low levels of methane and oxygen). However, it is precisely the acceleration of conversion of methane to CO_2_ that could dramatically reduce net radiative forcing and overcome the practical challenges associated with methanol production. Additionally, the ability to operate at low levels of methane and oxygen would expand the application space for the catalyst where it is most needed and across a spectrum of methane sources. Recognizing that this chemistry is desirable from the perspective of atmospheric methane abatement, we investigated copper-doped zeolites in methane oxidation at low temperatures and atmospheric gas compositions, seeking to provide an urgently needed tool to combat global climate warming.

## Methods

### Copper Mordenite Synthesis

Ammonium mordenite zeolite powder (5 ± 0.1 g; Alpha Aesar) was stirred with 0.05 M copper nitrate solution (500 mL) for 22–26 h and then vacuum-filtered through a glass fiber filter (0.7 μm GFF). Filtered solids were dried at 130 °C for 10–14 h, transferred to a glass vial, and stored in a desiccator until use. Importantly, we note that this preparation route is benign and strives to meet Green Chemistry^27−29^ principles: the ion exchange occurs at room temperature with minimized volumes and relies on earth-abundant, non-toxic materials, without acidic or organic solvents, with low energy requirements, and without the need for exotic or complex, multi-step syntheses. The process was highly reproducible.

### Reactor Design and Analytical Measurement

Gases were pre-mixed using a custom-built mass flow control array with electronic control, customized for the delivery of trace gases, including ultra-high-purity (UHP) helium, UHP oxygen, and 25, 700, and 70,000 ppmv methane in helium (Airgas). These were delivered to a vertically oriented, quartz tube furnace [16 × 1/2 O.D. inch (length × diameter)] fitted with a quartz frit and placed inside an Applied Systems 3210 series vertical tube furnace, which provided thermal control *via* an 850 W power supply. The reactor effluent was delivered to an SRI Instruments 8160C gas chromatograph with a flame ionization detector by direct injection every 90 s *via* two calibrated loops (5 mL each) and a Valco Instruments eight-port valve. The GC was calibrated daily with authentic standards (Mesa Specialty Gases).

### Catalyst Activation and Methane Conversion Reactions

In all cases, after loading 1 g of copper mordenite (approximately 3 cm height) into the vertical tube furnace, the reactor volume (34.5 mL) was flushed with helium while heating prior to introduction of activation gases. Then, experiments were conducted in one of two modes: a two-step activation-then-reaction or an isothermal reaction. For the “two-step” process, the catalyst was activated in an atmosphere of 20% oxygen and 80% helium (at 450 °C for 30 min unless otherwise noted) or 100% oxygen. Subsequently, the conversion reaction was carried out in the same atmosphere with additional methane (0.0002–2% methane; 30 min at 200 °C unless otherwise indicated). Note that traditional work on these types of materials for methane-to-methanol conversion relies on the two-step process to avoid overoxidation of methane to CO_2_. In the activation step, 100% O_2_ atmospheres are used to maximize efficacy, precluding simultaneous delivery of methane and necessitating segregated activation and reaction steps. In our experiments, we sought to simulate the operation in real air (*e.g.*, 20% O_2_ with methane). In all cases, we held the total flow of gas constant to preserve an approximately 30 s residence time of gas in the reactor. For the “isothermal” process, the catalyst was activated for 30 min (or 8 h for a long-duration study) under methane-free, 20% oxygen, and 80% helium and then reacted in 0.0002% methane for 30 min (or 300 h for a long-duration study) at a constant temperature. In order to achieve a constant temperature in the reactor, a continuous supply of heat was provided through a power supply with temperature feedback. Isothermal processes were explored to investigate the possibility of catalytic function without a thermal cycle (*i.e.*, duty cycle), which brings a net energy and operational expense savings during operation (*e.g.*, energy savings are conferred by avoiding heating and cooling cycles).

At the beginning of the reaction step, directly following the introduction of methane to the feed stream (70 sccm in total), a 15 min flushing time was allowed to ensure adequate mixing within the catalyst bed volume. After this equilibration phase, injections were made every 90 s for an additional 30 min (21 injections), and an average of these values (21 injections for the two-step reactions; five injections for isothermal reactions) was used to establish the effluent concentration. Standard deviations were less than 2.6% of the mean. Conversion efficiency was calculated by comparing the effluent methane concentration with the influent methane concentration (no catalysts, same gas mixture) at the same temperature. There was no evidence of non-catalytic methane oxidation (*i.e.*, direct combustion) at any temperature below 550 °C, and copper-free mordenite zeolite powder illustrated no potential to convert methane at the assayed temperatures (*i.e.*, 200 °C).

### Material Characterization

Copper-doped mordenite metal content was determined by inductively coupled plasma mass spectrometry (ICP–MS) using a NexION 300D. Briefly, samples were prepared by refluxing approximately 1 g of catalyst in 50 mL of 50% v/v HNO_3_ for 2 h until the solids were visibly bleached white. The leachate was extracted, concentrated to 10% of its initial volume, and then reconstituted in 2% HNO_3_. All copper loadings fell between 1 and 2 weight percent (see Supporting Information Figure S1). The crystal structure of the copper zeolite was studied by X-ray diffraction (XRD) using a PANalytical X’pert PRO diffractometer equipped with Bragg–Brentano geometry and Ni-filtered Cu Kα radiation (λ = 1.5418 Å, 45 kV, 40 mA); data were recorded in the range of 5–80 2θ. All results were consistent with the dominant crystal structure of the native zeolite. Scanning electron microscopy was conducted using a Zeiss Merlin Gemini field emission scanning electron microscope (5 kV accelerating voltage; 184 pA probe current) (Supporting Information Figure S2). A photograph of the catalyst before and after use shows a noticeable color change (Supporting Information Figure S3).

## Results and Discussion

### Activation and Reuse Potential

**Figure 1:**
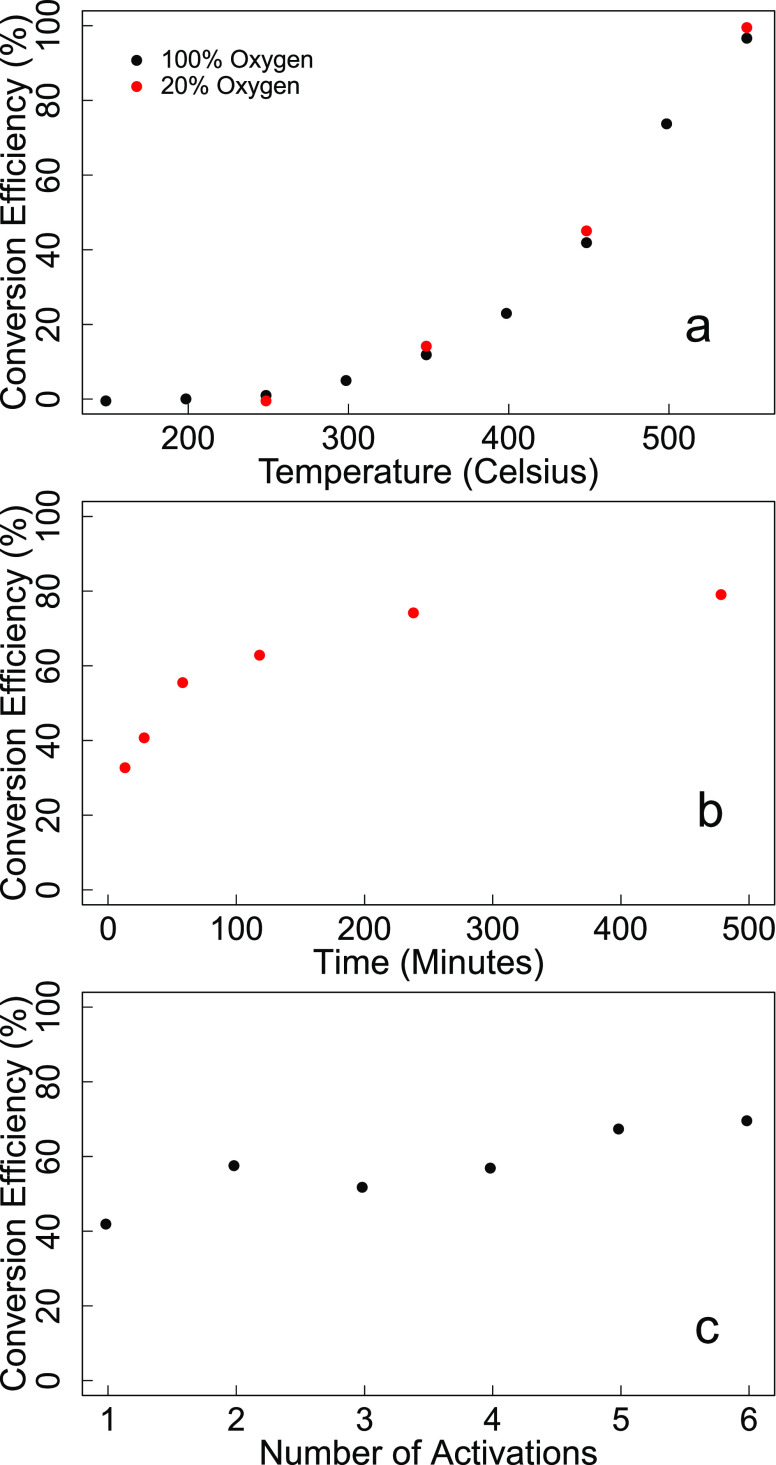
Catalyst activation as a function of gas composition, temperature, duration, and repetition illustrates that activation can be achieved under ambient-to-moderate conditions with reuse potential. The carbon conversion efficiency during the reaction steps is shown for all activation trials, which were conducted for 30 min at 450 °C and with a freshly prepared catalyst unless otherwise noted and in 100% (black) or 20% oxygen (red) in helium mixtures. All methane conversion reactions were conducted at 200 °C in 2 ppmv methane; note that 200 °C is not the maximum conversion temperature but enables one to see a spread in the behavior as a function of the activation temperature. The effect of (a) activation temperature, (b) activation duration, and (c) multiple reactivations is shown.

The performance efficiency of catalysts for methane capture and conversion is typically evaluated under a two-step process, where the first step (“activation”) occurs in methane-free, oxygen-rich conditions, and the second step (“reaction”) occurs in the presence of the reagent methane. Conducting all reaction steps at 200 °C allowed the evaluation of the activation step parameters spanning a range of thermal, temporal, and gas composition conditions. After relatively short (30 min) activations, increasing temperature in 100% oxygen improved reaction conversion efficiencies, where modest conversion efficiencies of 40% were observed at 450 °C, and 80–100% conversion was shown at 500 and 550 °C, respectively (Figure 1). These same efficiencies were observed in 20% oxygen atmospheres, indicating that the oxygen level was not limiting to the activation, and thermal effects may be more important. This has promising implications for field deployment of the catalyst as ambient oxygen levels (*e.g.*, from air) might be useful for catalyst activation, rather than requiring the presence of explosive levels of reactant oxygen. While the high end of observed activation temperatures and durations are within the range of previous studies (at or above 450 °C^15,16,18,20^ and with durations at or above 1 h^18,20^), use of ambient levels of oxygen has not been demonstrated previously for copper zeolites.^17^ Some novel “lean methane” catalysts based on cobalt,^30^ nickel mixtures,^31,32^ or platinum group metal (PGM)^33−38^ structures have been shown to work at or below 10% oxygen,^30,33,34,36,38^ but these require both high temperatures, expensive or complex synthetic processes, applied voltage, and/or a combination of activation gases far from ambient.

The impact of activation time was clear, where progressively longer 450 °C treatments conferred better methane conversion efficiency during the reaction. A short, 15 min activation resulted in only modest conversion, but this increased to over 60% by 2 h and nearly 80% after 8 h. While the conversion metrics of previous studies are not directly comparable, prior methane-to-methanol conversion attempts typically utilize oxidations of 1 h or longer.^16,18,20^ Extremely short or implicit activation times in a unique reaction environment have shown little reactivity (*e.g.*, heating to 270 °C in 1% oxygen, 3% water, and 18% methane; 0.03% conversion).^17^ In our work, because copper oxidation is kinetically fast, the time dependence suggests (1) that either gas transport through the constrained zeolite pore structure is slow or (2) that there is a critical, thermally mediated structural rearrangement of the copper zeolite or atoms therein that occurs slowly. While the former could be addressed by constructing hierarchical materials with improved gas access without compromising the pore structure, the latter might require re-engineering of the catalyst nano-structure or strategic thermal pre-treatments as part of the catalyst preparation.

**Figure 2:**
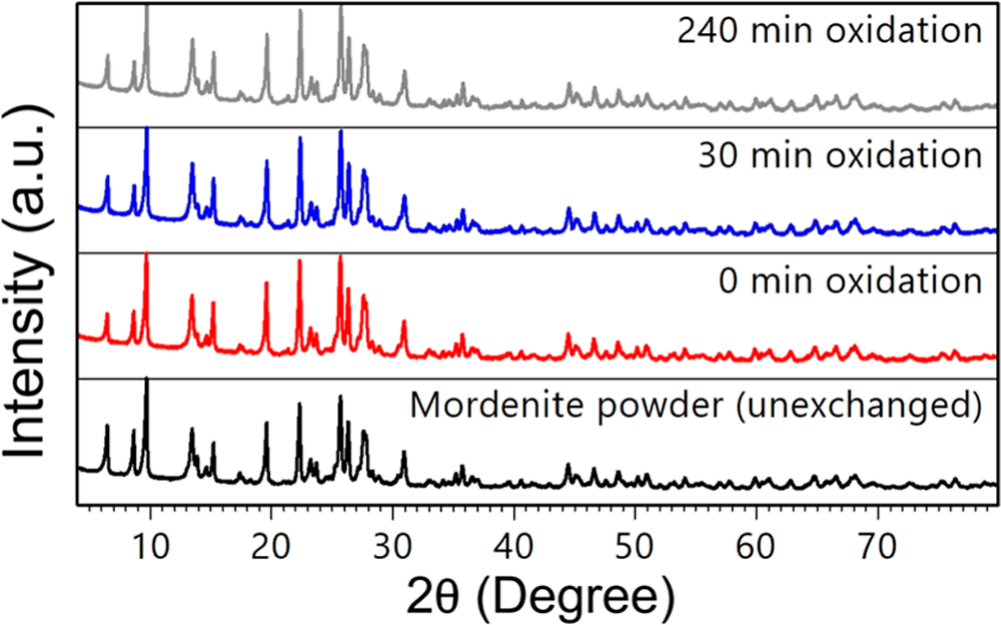
Powder XRD pattern of copper-exchanged ammonium zeolite powder (mordenite) after activations varying from 0 to 240 min in duration (unexchanged, 0, 30, 240 min from bottom to top).

To explore the possibility that longer-duration thermal pretreatments were giving rise to uniquely active catalyst nano- or mesostructures, we utilized powder XRD to probe the existence of a thermally driven catalyst rearrangement (Figure 2). As a whole, the XRD spectra were consistent with mordenite powder structures, and no quantifiable copper or copper oxide phase was observed [likely due to the low loading of Cu; ca. 1% (see Supporting Information Figure S1 and associated discussion of anticipated Cu-oxidation state dynamics during the reaction^39−42^)]. As thermal treatment time increased, deformations in peak patterns emerged at low diffraction angles (less than 10° 2θ). These peaks correspond to planes that bisect mordenite’s large channel (6.5 × 7 Å) pores lengthwise (110, 020, and 200), whereas smaller pocket pores (2.6 × 5.7 Å) were not affected. Specific evolutions found were that the integral breadth of the first and second observable peaks (corresponding to the 110 and 020 planes, respectively) increased and decreased in equal measure [approximately 0.01 rad. Note that the integral breadth (area of the peak divided by height) was employed because it is less susceptible to overinterpretations of changes in peak shape at low angles that would be associated with the extractable parameter full width at half-maximum]. As integral breadth is inversely proportional to the average crystallite thickness normal to the reflecting plane,^43^ these observations are consistent with a crystal structure development in the mordenite pore channels over time, where the channel became more compressed normal to the 110 plane and more expanded normal to the 020 plane with longer duration thermal treatments. Thus, the longer activation times appear to be associated with a micro-structural change in the larger pore structure, which leads to better methane conversion.

Such long *in situ* activation timescales would only be practical in field deployments if the catalyst could be recycled or activated and then reacted in series. Engineered strategies to achieve this type of cycling chemistry exist in the form of reactors that can be strategically cycled (in series or parallel), such as regenerative thermal oxidation, regenerative catalytic oxidation, or catalytic recuperative oxidizers, all developed for the conversion of volatile organic compounds (VOCs) in industrial waste streams.^44−49^ With this in mind, catalyst re-activation was illustrated for at least six cycles, showing a general trend of improvement consistent with the effect of longer activation times. This repeatability suggests that the total methane conversion potential per unit of catalyst may be sufficiently high to reduce the cost of CO_2_ equivalent capture below critical thresholds of 15–50 USD/ton. Importantly, these reuse experiments were conducted by oscillating between 450 °C activation in 100% oxygen and 200 °C reaction in 20% oxygen, which would necessitate undesirable life cycle costs associated with the duty cycle and risk associated with the use of concentrated oxygen. As such, we explored a broader range of reaction conditions and operation strategies.

### Methane Conversion Approach

**Figure 3:**
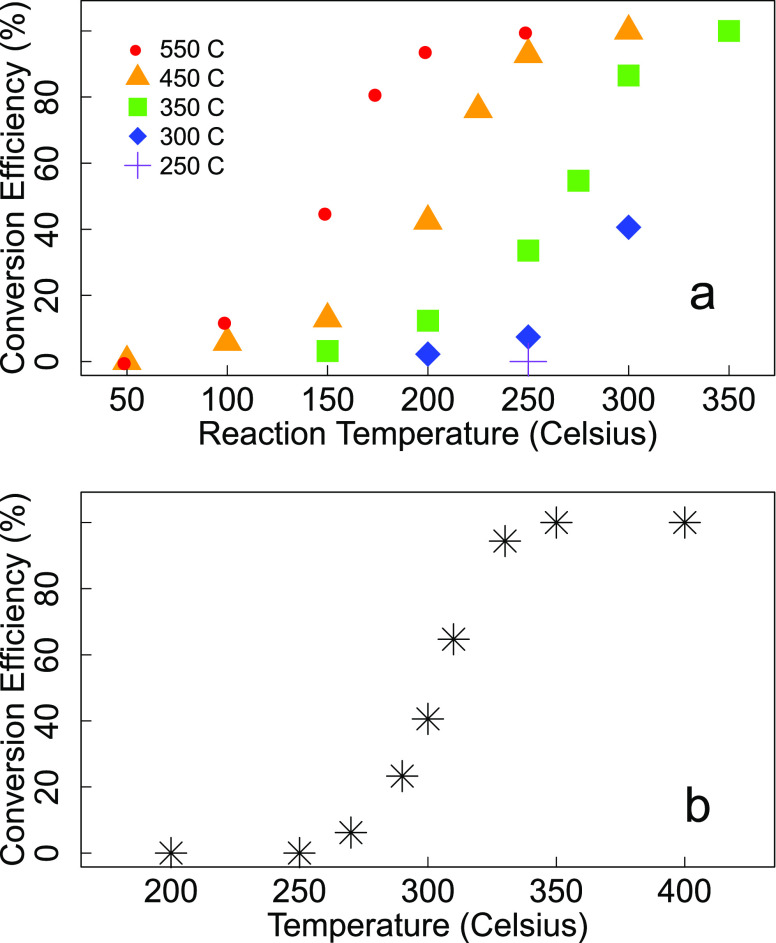
Methane conversion efficiency as a function of reaction temperature and operation mode. (a) In a two-step, activation followed by the conversion approach, both activation temperature and reaction conversion temperature influence methane removal. All activation steps were carried out for 30 min in 20% O_2_. (b) In a continuous reaction approach, 30 min activation in methane-free gas mixtures (20% O_2_) was followed by a 30 min conversion reaction in the presence of atmospheric levels of methane (2 ppmv) to simulate isothermal operations. Each data point was generated with a freshly prepared catalyst and represents the mean of at least 21 measurements.

Minimizing the temperature requirements of low-to-ambient level methane conversion reduces the energetic demand and infrastructural requirements of reactors, improves the overall operational lifecycle greenhouse gas benefit, and expands the possible deployment opportunities by reducing the flammability or explosion hazard (*e.g.*, for ventilation air at coal mines). To evaluate the potential for use at low temperatures, we varied conversion reaction temperature from 50 to 350 °C over a range of activation temperatures between 250 and 550 °C. At high activation temperatures (above 450 °C), non-zero catalytic activity was achieved at reaction temperatures as low as 100 °C, and over 40% conversion was achieved at 200 °C and above (Figure 3). At lower activation temperatures (350 °C or below), conversion over 40% requires systematically higher reaction temperatures (above 250 °C). Limited but quantifiable conversion was observed at temperatures as low as 100 °C (Figure 3a), reduced from previous experiments carried out between 150 and 200 °C. Catalytic conversion of methane using PGMs,^33−38^ cobalt,^30^ or nickel–cobalt mixtures^31,32^ has typically relied on higher activation temperatures (500–1000 °C) or long durations (*e.g.*, 24 h at 350–400 °C^33,35^); that is, successful activations generally exceed the time and temperature ranges explored here. Using relatively aggressive activations, successful conversions of 1000–10,000 ppmv methane (0.1–1% CH_4_, with 10% or less oxygen) at temperatures well below the ignition point (600 °C^50^) have been recorded^31,33,34,36,38^ with a few demonstrating conversion temperatures as low as 300 °C.^32,35^ While the reaction temperatures we observed are moderately lower than previous demonstrations, the dramatically different activation procedures and simplified catalyst synthesis offer unique benefits. As a point of comparison, conversion efficiencies to CO_2_ are not often reported and cannot be directly compared to our results; nevertheless, methanol production rates in previous studies have been quite low (order 0.1–0.3 mol methanol per mol Cu or less;^26^ methanol production was not systematically quantified in our study, but early spot checks in the two-step process revealed around 1–2 orders of magnitude lower methanol in the post-reaction, water-extracted catalyst, where the input methane was around 10^6^-fold lower than in previous studies). While CO_2_, formaldehyde, formic acid, and methanol are the only anticipated products in the oxidation pathway, we have not evaluated the potential formation of other products to date. To our knowledge, no prior studies have shown complete conversion of methane at these temperatures in simulated air with an easy-to-produce, earth-abundant catalyst.

The achievement of harmonized catalyst activation and reaction temperatures confers important operational and life cycle advantages. Specifically, the isothermal operation minimizes the need to repeatedly deliver power for heating the thermal mass of a reactor and the associated catalyst, reducing the levelized cost, energy, and greenhouse emissions. Using the operational space as a roadmap (Figure 3a) for optimizing catalytic efficiency, we demonstrated that the isothermal reaction offers complete removal of atmospheric methane at 350 °C (Figure 3b). Minor conversion (approx. 7.1%) was initially detected at 270 °C. This is consistent with the novel continuous reactions conducted by Dinh *et al.*,^17^ who demonstrated methanol production at this temperature, albeit with efficiencies below 1% (by design, to minimize “overoxidation” to CO_2_ and in dramatically different conditions). At modestly higher temperatures (*e.g.*, 300–310 °C), 42–67% of methane was oxidized following a 30 min activation. This is valuable for some remediation applications and could be pushed to higher conversion rates under different activation schema (see below). Levelized life cycle budgets that consider the potential trade-off between total methane abatement *versus* operational energy demands (both in CO_2_ equivalents) must be conducted to determine if the additional conversion merits the operational temperature. Theoretically speaking, the conversion of methane to CO_2_ is exothermic, and depending on the reactor design and flowrates, higher input concentrations of methane might be able to *generate* excess heat. For example, ventilation air in mines can contain between 0.1 and 2% methane.^51^ A simple calculation of energy generation compared to energy requirements can be derived from the theoretical energy generated by the reaction relative to the energy needed to heat incoming air to the operating temperature (eq 1).
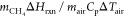
1where *m*_CH_4__ is the mass of methane in the incoming air stream, Δ*H*_rxn_ is the enthalpy of methane oxidation to CO_2_ (890 kJ/mol), *m*_air_ is the mass of incoming air with specific heat, *C*_p_ (700 J/kg K), and Δ*T*_air_ is the temperature change required to get from the ambient temperature to the operating temperature (*e.g.*, 310 °C). At these sources, a methane concentration of 1% gives a heat-generation-to-heat-demand ratio of 1.5; that is, the process *generates excess energy.* At the ventilation air flowrates used at mines (approximately 100,000–1,000,000 CFM), this could potentially yield electricity at the power-plant scale (order 5 kW, depending on specifics of the system). One could use this excess energy to pre-heat incoming air, offset the energy demands of the system (*e.g.*, “parasitic power” associated with air movement across a pressure drop), and ultimately drive electricity-generating heat exchangers to meet other needs on-site. Notably, such a system could accelerate payback times for the device as well. Considering that low-level methane sources responsible for the majority of emissions to the atmosphere span 4 orders of magnitude in concentration, it is possible and somewhat remarkable that this catalyst could be deployed with a net energy yield in modestly elevated methane scenarios (over approximately 0.67% methane; see Supporting Information Figure S4).

### Methane Abatement at All Sub-Flammable Thresholds

**Figure 4:**
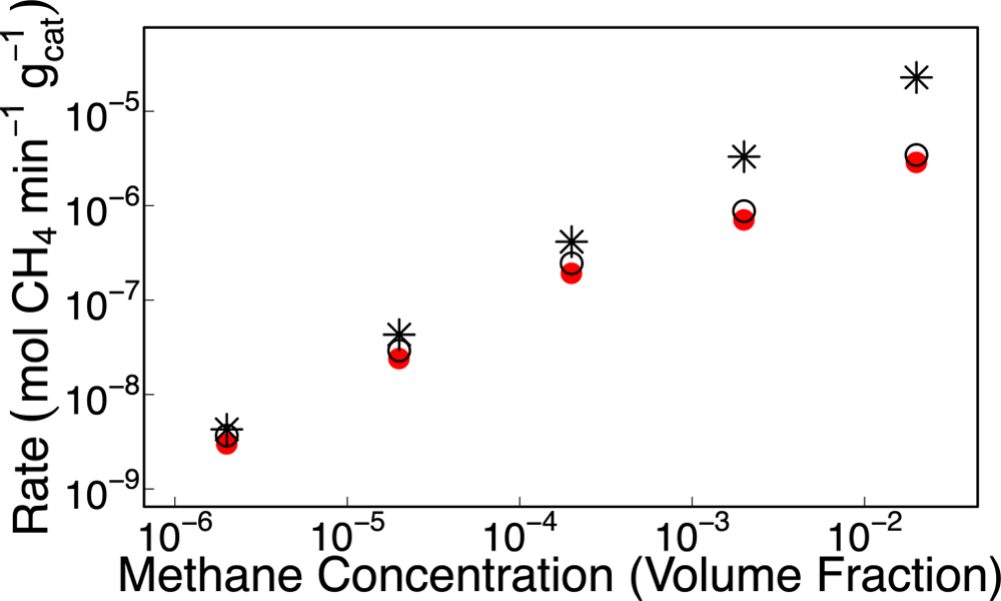
Methane conversion rate increases with the input methane concentration over a range of sub-flarable levels. Methane conversion was tested from 2 ppmv to 2% v/v methane in the presence of 20% oxygen in isothermal operation at 310 °C (30 min initial activation in methane-free gas; asterisks), and following 30 min (filled red symbol) and 60 min (open symbol) activations (450 °C) and reaction (200 °C) in 20% oxygen. Each data point collected with the freshly prepared catalyst represents at least 20 methane conversion measurements.

To explore the possibility of deploying the catalyst for methane abatement at any sub-flammable level, we evaluated incoming methane conversion rates between 2 ppmv and 2% v/v methane (*e.g.*, from near-atmospheric to typical ventilation air levels observed in coal mines). Overall, higher incoming methane corresponded with higher rates of conversion (Figure 4; 4.28 × 10^–9^ to 2.28 × 10^–5^ mol min^–1^ g_catalyst_^–1^ isothermally) but came at a cost to the total proportion of methane removed (Figure S5). Further, the conversion rate exhibited sensitivity to the activation time and temperature: isothermal reactions at 310 °C exhibited steady and monotonic increases in the methane conversion rate, whereas step-wise reactions at short and long (30 and 60 min at 450 °C) activations followed by lower temperature reactions (200 °C) showed a diminishing conversion rate with higher methane loadings. The influence of thermal history (*i.e.*, duration of elevated temperature treatment in both continuous and non-continuous modes) implies that there is a physical limitation to catalyst activation, consistent with our earlier observation of the importance of activation time (Figure 1). Promisingly, there does not appear to be a definitive ceiling in the catalyst’s ability to convert methane at higher concentrations, implying that higher conversion rates could be achieved *via* optimization of activation parameters and/or reactor geometry. Hierarchical material construction, such as preserving the nanoconfinement of the Cu-aluminosilicate active sites but supporting those on a substrate to promote better gas-catalyst contact, improved heat transfer properties by varying the material choice, and novel reactor design are all viable routes to enhance conversion rates. This potential for improvement aside, for the first time, these results demonstrate that copper mordenite can convert methane at low-level concentrations previously untested by either other zeolites, lean combustion, or ventilation air methane catalysts. Considering that these conversions can be achieved at any methane concentration of concern well below flammability limits (less than 5%) or ignition temperatures, strategic deployment at or near elevated methane sources would bring rapid benefit to reduce or reverse the exponential accumulation of atmospheric methane observed since the early 1800s.^5,52−54^

### Catalyst Conversion Capacity and Lifetime

**Figure 5:**
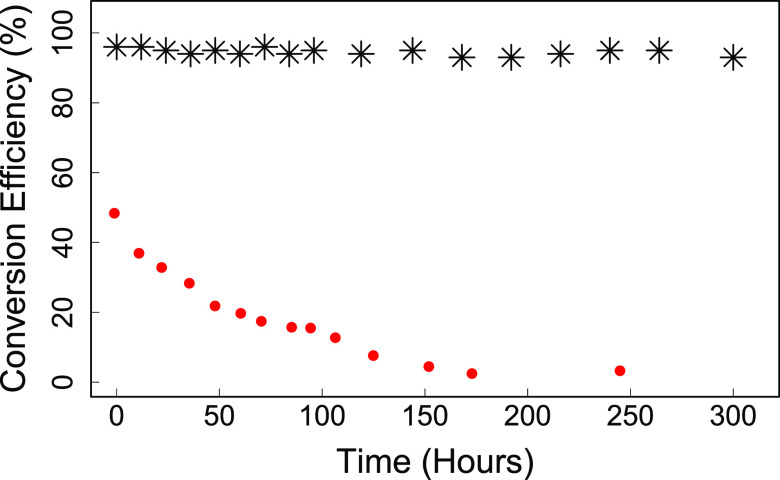
Long-term activity of the catalyst. Low-level methane (2 ppmv methane in 20% oxygen) was catalytically reacted over 300 h under continuous, isothermal operation at 310 °C (asterisks, following 8 h activation) and a traditional two-step process (red circles; 450 °C, 30 min activation followed by 200 °C continuous reaction).

Viability and adoption of this catalytic technology will depend on both the opportunity for levelized greenhouse gas reductions and the total cost of the abatement strategy. Both scale with the reusability of the catalysts and requisite strategy for regeneration (*e.g.*, thermal cycling or chemical recharge). First, a traditional two-step activation followed by a reaction at differential temperatures illustrated a relatively rapid deterioration in the methane conversion potential (30 min, 450 °C activation in 20% oxygen followed by continuous exposure to 200 °C, with 2 ppmv methane added; Figure 5). In contrast, a pre-activation for 8 h followed by isothermal reaction at 310 °C showed prolonged, near complete methane removal for up to 300 h (12 days). The high reactivity initially was a consequence of the long activation time and consistent with earlier observations (Figure 1b) that activation time promotes formation of reactive pore structures observed by XRD (Figure 2). Maintenance of a strong catalytic activity over this time suggested that either the catalyst capacity was never reached or that the catalyst was reactivating and performing in a continuous manner (*i.e.*, regenerating itself, as in the definition of a catalyst). The dramatically different behavior observed between a two-step activation and an isothermal operation is the outcome of both thermal and kinetic effects. In a two-step process, the short, high-temperature (450 °C) activation is followed by operation at a relatively low temperature (200 °C). The continuous decay in performance implies that 200 °C is insufficient to reactivate and fails to maintain the activity of the catalyst. The low conversion efficiencies (approaching 0% methane conversion) are consistent with low-temperature activation experiments (see Figure 3a for 250 °C activation and reaction). In contrast, a long activation step (8 h) at 310 °C gave a high and consistent methane conversion performance. The near complete conversion of methane (100%) exceeded the conversion at 300 or 350 °C for only 30 min (40–50% methane conversion; Figure 3a), reinforcing the sensitivity of catalyst performance to activation time (see Figure 1b) associated with catalyst pore re-structuring (Figure 2). While the long-duration, 8 h activation could initially produce a very reactive catalyst structure that is maintained or regenerated, it is also possible that the isothermal reaction condition is functionally serving as a very long “activation” in and of itself, conferring sustained catalytic activity.

Continuous reactivity under isothermal operations increased the overall quantity of converted methane more than fivefold compared to the two-step reaction (0.3 *vs* 1.7 mg CH_4_ g_catalyst_^–1^ total when integrating over the duration of the long-term trials). Here, we note that these conversion ratios are only relative to one another and cannot be used to determine the total potential of the catalyst for methane drawdown from the atmosphere or near more concentrated sources. Indeed, evidence suggests that the total conversion capacity may be much higher if larger input methane concentrations were delivered (Figure 4).

### Implications, Novelty, and Feasibility

The application of zeolites for biomimetic methane conversion has been explored for decades, but several key limitations existed in prior work that obfuscated the utility of these catalysts for low-level methane abatement. First, there had been a focus on stopping the reaction at methanol, so that methanol could be used as a chemical feedstock. However, technical challenges existed that prevented the arrest of the reaction at methanol (which proceeds exergonically to CO_2_) and separation of the liquid stream from the gaseous input. Further, current methanol markets (75 MMT in 2018) would only abate about 37.5 MMT of methane, enough to reverse atmospheric accumulation but insufficient to offset major atmospheric source terms and restore atmospheric methane levels quickly. Second, methanol separation is only technically feasible at the most concentrated methane input streams but remains uneconomic due to the relatively low methanol yields. Methanol is often recovered and measured *via* water extraction of the catalyst, which would create major technological hurdles in and of itself. Third, in an effort to avoid “over oxidation” to CO_2_, a practice emerged in which zeolites were cycled between oxygen-rich and methane-rich environments (*i.e.*, the activation and reaction steps, respectively). The key novelty of our work is (1) the demonstration that catalyst turnover can be achieved in air at modestly low temperatures, in a continuous fashion, and at exceedingly low levels of methane in the sub-flammable region and (2) the recognition CO_2_ is the desired product, overcoming separation challenges while conferring major climate-impact reduction. Any concern for CO_2_ generation as a pollutant is misguided: methane-to-CO_2_ conversion brings an instantaneous radiative forcing reduction and converting 60% of the atmospheric methane reservoir (*i.e.*, restoration to pre-industrial levels) would result in a modest 1.1 ppmv increase in CO_2_ (*e.g.*, from 415 ppmv CO_2_ today to 416 ppmv CO_2_).

The ability to mitigate low-to-ambient levels of methane in simulated atmosphere indicates promise to deploy a stand-alone system anywhere where fugitive methane emissions exist. However, maximum environmental impact and economic yield (*i.e.*, greenhouse gas (GHG) equivalents removed per $) would come with strategic deployment at relatively enriched methane sources (*e.g.*, dairy and meat barns or coal mines). We described the potential for low or no energy needs on-site depending on the input methane concentration and the associated thermal yield. At high methane sources (*e.g.*, coal and mineral mines, where ventilation air can be up to 2%), excess heat can meet the demands of heating incoming air and the catalyst and even offer excess electricity generation (see Supporting Information Figure S4). This energy generation potential could improve the net GHG benefit of the technology. If modest carbon taxes are instated, the energy generation coupled with the sale of superfluous electricity could potentially pay back capital equipment expenses and operating costs over a relatively short time (order decade). Here, we note that the catalyst is synthesized from earth-abundant Cu and clay aluminosilicates. We estimate the copper-zeolite catalyst costs to be on the order of *cents per pound-* $0.15–0.82/lb, many orders of magnitude lower than those of competing technologies. Thus, there is great potential for this technology to be developed at low cost and with minimum environmental impact, potentially reducing operating costs below critical thresholds of proposed CO_2_ pricing strategies (*e.g.*, below $15–50/ton of CO_2_ equivalents).

Several technical achievements remain on the path to feasible deployment. Catalyst poisoning regimes need to be tested with typical atmospheric interferents (primarily water) and other light VOCs that might prematurely saturate or spoil the catalyst. Pre-filtration strategies may be needed to overcome any emergent complications, and real-world transformation products should be monitored on initial deployment. Ideally, control systems and *in situ* monitoring capability would ensure the continuous function and efficacy of any commercial device. Finally, the catalyst material should be supported or structured in a way such as to maximize air flow through the reactor system. Then, reactors could be interfaced downstream of extant air handling capacity on-site and further lower the levelized GHG impact and cost.

One such integration would be with ventilation air systems at coal mines. Coal mining disasters prompted the creation of the 1977 Mine Safety and Health Act and 2006 MINER Act in the US. Associated Mine Safety and Health Administration (MSHA) standards, while necessary to protect miners, present technological challenges to methane abatement in ventilation air, as explosive or hazardous reagents must be avoided. As such, state-of-the-art mine ventilation air systems offer zero methane conversion. Here, we demonstrate low operation temperatures and alleviation of the need for oxidative reagents, improving compatibility with MSHA guidelines. Incorporation of our catalyst to ventilation air systems could mitigate nearly 39 MMT of CO_2_ equivalents (CO_2,e_) in the US,^55^ translating to nearly 410 MMT of CO_2,e_ if deployed globally.^48,55,56^ Atmospheric methane levels are growing on the order of 16.8 MMT methane annually^5^ (470–571 MMT CO_2,e_ using methane’s 100-year global warming potential range of 28–34^3,4^). Thus, if scaling challenges are addressed, the proposed technology could nearly offset the trend of accumulating atmospheric methane, offering critically needed near-term climatic forcing benefits.

Jackson and colleagues highlighted atmospheric restoration to pre-industrial methane levels (750 ppbv *vs* today’s 1860 ppbv and steadily climbing CH_4_) as a mechanism for saving 16% of global climate forcing.^7^ This near-term impact would be felt within decades, whereas CO_2_ mitigation strategies would not exert influence for 50 years or more.^11^ While both are necessary and urgently needed, efforts to address near-term warming GHGs in the atmosphere provide a unique route to stall the effects of anthropogenic warming, providing much needed time to adapt and respond to the changing climate.

## Supporting Information Available

Additional material characterization details, including ICP analysis, SEM images, and photographs of the catalyst (PDF)
