# Supplementary material for: Atmospheric- and Low-Level Methane Abatement via an Earth-Abundant Catalyst
Source: ACS Environ Au. 2021 Dec 29;2(3):223–31. doi: 10.1021/acsenvironau.1c00034 (PMC10114903; doi:10.1021/acsenvironau.1c00034)
Supplement: Supplementary file 1 — vg1c00034_si_001.pdf [file vg1c00034_si_001.pdf]

Supporting Information for:
Atmospheric- and low-level methane abatement via an Earth-abundant catalyst

Rebecca J. Brenneis[1], Eric P. Johnson[1,2], Wenbo Shi[1], Desiree L. Plata[1*]

[1]Ralph M. Parsons Laboratory, 15 Vassar Street, School of Engineering, Massachusetts Institute of Technology, Cambridge MA
[2]17 Hillhouse Ave, School of Engineering, Yale University, New Haven, CT

*corresponding author email: dplata@mit.edu

Elemental analysis

Copper contents of ion exchanged zeolite (Mordenite) were determined using inductively coupled plasma mass spectrometry (ICP-MS). Copper zeolite powder (0.24-0.26 g) was leached in 20 mL of 100% v/v nitric acid over a hotplate and refluxed for 1 hour. At this point, the solution was uncovered and allowed to vaporize until less approximately 1 mL of liquid remained. The remaining liquid filtered with a 0.22-micron filter, and reconstituted to 50 mL with a 2% nitric acid solution. Solutions were spiked with a multiple component internal standard (Multi-element Calibration Standard 3 by Perkin Elmer). Five-point calibration standards were made with a 1,000 ppm copper standard in 2% nitric acid. Samples of copper zeolite were collected after ion exchange times varying from 0.25 to 7 days and the copper loadings reached equilibrium after 6 hours.

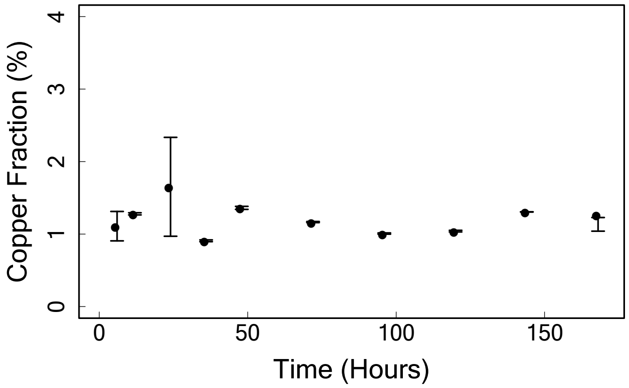

Figure S1. Copper loading of the catalyst. Samples with varied ion exchange times (0.25-7 days) were analyzed to determine whether copper loading increased with time, and results indicated that steady-state Cu concentrations were reached after only 6 hours of stirring.

Scanning Electron Microscopy

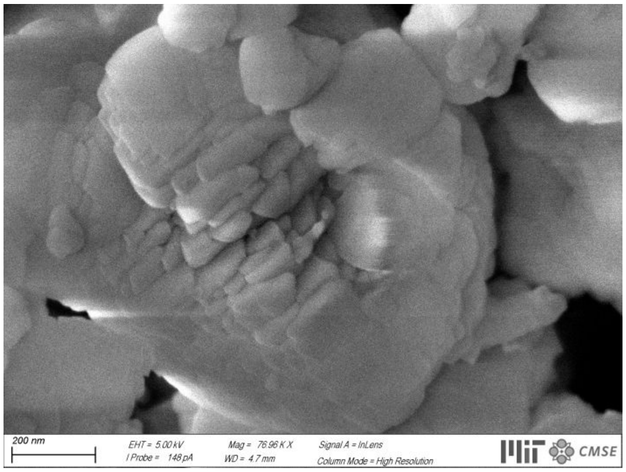

Figure S2. Scanning electron micrograph of copper zeolite (ZSM-5) is unremarkable and shows common clay-like structure.

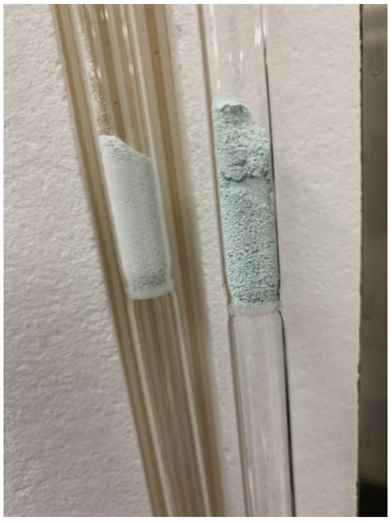

Figure S3. Photograph of catalyst in vertical tube furnace before activation (left) and after reaction (right).

Outstanding needs for characterization of copper dynamics
Changes in the coppoer oxidation state were expected but not readily imaged or analyzed in this contribution due to the low copper loading in the internal zeolite pores and other limiting factors. First, the reaction should necessarily proceed through an oscillating Cu oxidation of 1 or 2 electron equivalents, and we anticipate that the oxidation state would cycle as the catalyst turns over during the reaction in air. However, analyzing such a change in copper oxidation state is notoriously difficult due to the rapid oxidation of copper in air (O'Connor et al.; Kim et al.). For example, x-ray photoelectron spectroscopy (XPS) interrogates the surface of a material only (~ 10 nm), and even on a reduced Cu, if the Cu surface becomes oxidized (which it quickly does at room temperature in air), a mixed Cu-oxide state would be observed. Since we are actively treating our Cu at 300°C and above in air, it would appear fully oxidized (i.e., as Cu^{2+}) in any *ex situ* analysis. As such, to observe the reaction dynamics, an ideal approach would invoke the use

of *in situ* or "environmental" scanning electron microscopy (SEM), in which the local atmosphere of the reaction can be controlled, coupled with XPS or an energy loss technique to develop a sense of the oxidation state of the metal. The conditions of the reaction (atmospheric pressure and low-level methane in air at 300°C) make *in situ* monitoring challenging, because SEM requires operation at vacuum (around 10^{-4} Torr at a minimum). Nevertheless, the feat has been achieved previously for similar heterogeneous catalysis reactions (Steiner et al.; Hofmann et al.), and such approaches could be pursued in subsequent study of this important catalyst.

Theoretical calculation of energy demand relative to energy generation potential.

A simplified calculation of energy generation compared to energy input requirements can be derived from the theoretical energy generated by the reaction relatively to the energy needed to heat incoming air to operating temperature (Eqn 1).

$$m_{CH4}\Delta H_{rxn}/m_{air}C_p\Delta T_{air}, \qquad \textbf{Eqn 1}$$

where m_{CH4} is the mass of methane in the incoming air stream, ΔH_{rxn} is the enthalpy of methane oxidation to CO_2 (890 kJ/mol) [Prosen and Rossini, Rossini, Cox and Pilcher, Roth and Banse; all available in the NIST Webbook accessed Dec 13, 2021], $m_{air}$is the mass of incoming air with specific heat, C_p (700 J/kgK) and ΔT_{air} is the temperature change required to get from ambient temperature (e.g., 20°C) to operating temperature (e.g., 310°C).

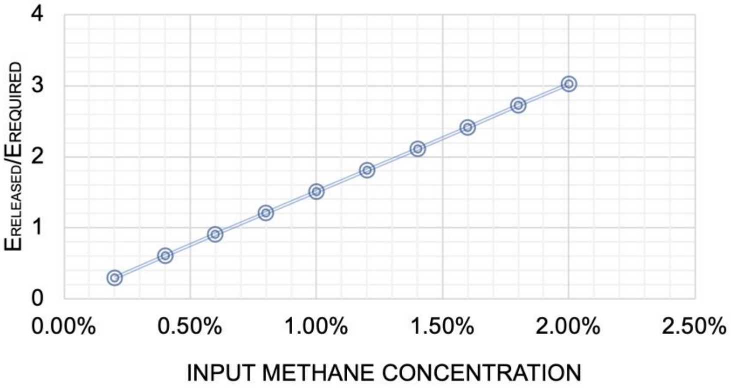

Figure S4. Theoretical thermal yield as a ratio of heat generated by the catalytic conversion of methane to CO_2 relative to the heat required to heat incoming air. The calculation assumes that air needs to be heated from 20°C to 310°C.

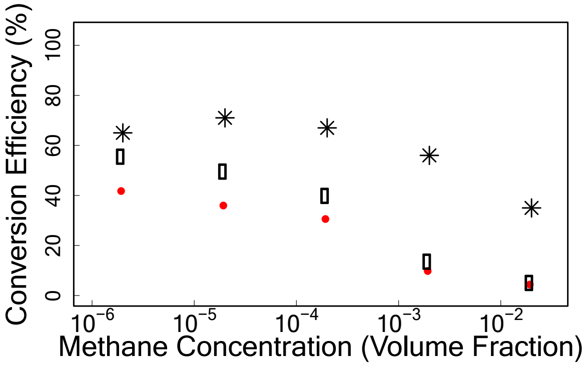

Figure S5. Conversion efficiency of methane removal over a range of sub-flareble levels. Methane conversion was tested from 2 ppmv to 2% v/v methane in the presence of 20% oxygen in isothermal operation at 310°C (30-min initial activation in methane-free gas; asterisks), and following 30-min (filled red symbol) and 60-min (open symbol) activations (450°C) and reaction (200°C) in 20% oxygen.
